# Systematic preoperative approach for bariatric surgery, perioperative results, and economic impact

**DOI:** 10.3389/fpubh.2024.1439948

**Published:** 2024-10-09

**Authors:** Iolanda Freire-Moreira, Maria Pilar Sanchez-Conde, Gilles Barreira-de Sousa, Maria Isabel Garrido-Gallego, José María Rodríguez-López, Raúl Juárez-Vela, Juan Alonso Bragado, Marta Carretero-Hernández, Carlos Ricardo Vargas-Chiarella, Jesús Calderón-Moreno, María Fernanda Lorenzo-Gómez, Luis Mario Vaquero-Roncero

**Affiliations:** ^1^Department of Anesthesia & Intensive Care, Salamanca University Complex, Salamanca, Spain; ^2^Faculty of Medicine, University of Salamanca, Salamanca, Spain; ^3^Department of Cardiology, Salamanca University Complex, Salamanca, Spain; ^4^Faculty of Health Sciences, University of La Rioja, Logroño, Spain; ^5^Department of Business Economics, Applied Economics, and Fundamentals of Economic Analysis, Rey Juan Carlos University, Madrid, Spain; ^6^Department of Urology, Salamanca University Complex, Salamanca, Spain

**Keywords:** cost control, economics, anesthesia, surgery, effectiveness

## Abstract

**Introduction:**

Obesity is a complex systemic condition, involving numerous anatomical and metabolic changes. Therefore, a comprehensive preoperative assessment is essential for each patient contemplating bariatric surgery.

**Objetive:**

This study presents the findings of a proposed protocol designed to streamline the pre-anesthesia consultation process. Our aim was to compare the efficiency and costs of consultations guided by the protocol with those conducted without a specific strategy. The secondary outcomes assessed included postoperative (PO) length of hospital stay and surgical duration.

**Matherial and methods:**

We conducted a retrospective cross-sectional analysis involving 206 clinical cases. Statistical analyses, including the chi-squared test, Student’s t-test, and Mann–Whitney U test, were utilized based on the type of variables.

**Results:**

The results showed a significant reduction in the costs, pre-anesthesia consultation duration, time spent in the recovery unit, and the need for referrals. However, no statistically significant differences were observed in the delay before surgery and length of hospital stays, measured in days.

**Conclusion:**

This algorithm offers a promising approach for optimizing perioperative management in bariatric surgery, demonstrating its effectiveness in cutting costs and reducing the need for referrals.

## Introduction

Obesity is considered a multifactorial systemic disease, defined by the World Health Organization as having a body mass index (BMI) value equal to or greater than 30 kg/m^2^ (1, 2). However, this definition has been questioned by entities, such as the European Association for the Study of Obesity, due to its inability to differentiate between muscle mass and adipose tissue, the latter being more related to morbidity (3, 4). Despite these concerns, BMI remains the most commonly used tool due to its good correlation with other obesity parameters and its ease of interpretation. When abdominal circumference is considered together with BMI, the prediction of individual metabolic risk improves, given its stronger correlation with visceral fat distribution (5).

The economic impact of obesity is increasingly negative, manifesting itself in higher healthcare budgets, higher unemployment rates, losses in gross domestic product (GDP), and reduced life expectancy (2, 6–9). Studies have shown that obesity is directly related to higher healthcare costs, especially among women in their 60s and 70s, and it results in as much as a 30% increase in healthcare expenses across all age groups compared to non-obese individuals (10, 11). Since 1980, the global obesity and overweight rates have doubled, affecting up to one-third of the world’s population, exacerbating healthcare burdens and population morbidity and mortality rates, both from medical conditions and during surgical interventions (12–14).

To address perioperative risk in obese patients, the European Society of Anaesthesiology and Intensive Care (ESAIC), the American College of Cardiology (ACC), and the Royal College of Anaesthetists (RCoA) have formulated specific guidelines (5, 15, 16).

The Global Burden of Disease Project observed a J-shaped risk relationship between BMI and all-cause mortality, with higher mortality rates both below 20.0 and above 25.0 kg/m^2^ compared to the 20.0–25.0 range. In this study, the all-cause mortality rate was found to be 7% for individuals with a BMI between 25.0 and 27.5 and 20% for those with a BMI between 27.5 and 30.0. Additionally, the attributable risk for grade 1, grade 2, and grade 3 obesity was 45, 94%, and up to 176%, respectively. Whitlock et al. found that for every additional 5 units increase in BMI, there was a 30% increase in overall mortality, a 60% rise in mortality due to chronic kidney disease, and a 120% increase in mortality related to diabetes mellitus (DM) (17).

Bariatric surgery refers to surgical procedures aimed at weight loss, currently targeting patients with a BMI value >40 or > 35 and a comorbidity or failed medical treatment; the latter criteria have a subjective connotation as no clear cross-sectional consensus has been reached (2, 18). Among the available approaches, tubular gastrectomy (TG) is the most common, followed by Roux-en-Y gastric bypass (RYGB), as the demand for gastric bands and balloons has declined due to poor long-term results; all approaches aimed to achieve weight loss, either by limiting food intake capacity or inducing malabsorption (2). In patients with type 2 DM, TG has gained popularity over RYGB despite resulting in less weight loss and DM remission. This is because TG results in fewer postoperative (PO) complications, shorter hospital stays, and lower costs, and it has even gained international support for an earlier surgical approach (i.e., poorly controlled DM + grade 1 obesity) (18–20). The Longitudinal Assessment of Bariatric Surgery (LABS) reported a 30-day mortality rate of 0.3% and a long-term complication rate of 2% at 1 year (21, 22). Bariatric surgery is not recommended for patients with acute coronary syndromes, those with severe non-responsive obstructive sleep apnea/hypopnea syndrome (OSAHS), those who cannot understand the procedure or comply with PO restrictions, and those with continued drug abuse or a malignancy that limits expected survival to less than 5 years (23).

Pre-anesthetic assessment is a fundamental clinical process to ensure patient safety during anesthesia administration, surgery, and the postoperative period. The aim is to understand the patient’s baseline conditions and comorbidities in order to anticipate potential complications, estimate the perioperative risk, and prepare an optimization plan that will help the patient attend the procedure in the best possible condition; emphasis is placed on homeostatic disturbances due to anesthesia and surgery. Therefore, a thorough assessment provides a greater benefit in bariatric surgery when considering the above-mentioned implications of obesity (23, 24).

OSAHS is associated with an increased risk of respiratory depression due to the effects of opioids and sedatives, airway obstruction with facemask ventilation, and difficulty with laryngoscopy/intubation. Surveys can help to better assess these risks, such as the risk score for obstructive sleep apnea—STOP-Bang questionnaire, which has been most widely studied for detecting OSAHS and predicting preoperative adverse effects due to its easy application and high sensitivity (25, 26). Disease severity and treatment should be assessed, and a continuous positive airway pressure (CPAP) regimen should be maintained until surgery and reintroduced during the immediate postoperative period. This is because CPAP reduces tongue volume, increases pharyngeal space, improves blood pressure control, and reduces hypoxemic events and polycythemia (15, 27–29).

Neck circumference, especially if >102 cm, correlates better than the Mallampati score with the Cormack–Lehan scale of direct laryngoscopy and difficult intubation (DI) (30, 31). Cardiovascular disease has a linear relationship with BMI, so a thorough assessment is required, including a 12-lead electrocardiogram if there is a history of diabetes mellitus, smoking, hypertension, hyperlipidemia, or poor exercise tolerance (5, 32, 33). In addition, special attention should be paid to hypertension as it is associated with cardiac, neurological, and renal complications; therefore, existing guidelines recommend postponing non-urgent surgery to improve treatment when systolic blood pressure values >180 mmHg or diastolic blood pressure values >110 mmHg are found (15). If necessary, blood glucose levels, nutritional deficits—especially folic acid and iron metabolism—thyroid hormones, and renal function should also be measured and optimized before bariatric surgery (15, 34–37).

It is advisable to combine validated assessment tools for pre-anesthesia, including classic tools, such as the American Society of Anesthesiologists (ASA) status or New York Heart Association (NYHA) Functional Classification, and more recent tools, such as the Revised Cardiac Risk Index (RCRI), the National Surgical Quality Improvement Program (NSQIP), the Obesity Surgery Mortality Risk Score (OS-MRS), the Metabolic Equivalent of Task (METS), and the Gupta Perioperative Cardiac Risk Calculator (Gupta) (38–40). The NSQIP and the Gupta tools, which are based on patients’ data, predict risks of complications and the length of stay, having proven useful in reducing morbidity, mortality, longer stays, and hospital-related costs, while the RCRI can help distinguish healthy obese patients who do not require further cardiac evaluation from those who might benefit from a consultation with a cardiologist, thereby saving time and resources (38, 41). The OS-MRS is specific to bariatric surgery, allowing clearer information to be provided to the patient regarding mortality risk (24). The METS, which are derived from patient responses, may be underestimated by up to 3.3 units; however, it remains a proven tool for assessing patient suitability as the American College of Cardiology advises against major elective non-cardiac surgery in patients who cannot achieve 4 METs (39, 42).

Clinical algorithms have been in use for some time and can reduce human error, improve efficiency and cost-effectiveness and pave the way for future research, but they also present challenges, such as health professionals’ reluctance to implement them, which can be addressed by the legal support these algorithms provide (15, 36, 37, 43, 44).

Currently, there is no standardized pre-anesthetic assessment algorithm for patients with obesity, although specific scientific recommendations exist (5, 15, 16). Obesity itself is considered a high risk, leading to overly extensive testing and patient referrals, which results in greater costs and unnecessary delays in surgery (15, 45–49). Algorithms and clinical pathways can help reduce costs and improve the quality of care while maintaining or improving patient safety (50).

In the aforementioned scenario, we conducted a thorough examination and established a protocol for addressing the systematic approach to morbidly obese patients during pre-anesthesia assessment. Our core aim was to compare the cost and timeline of algorithm-guided consultations with those conducted without a specific protocol. The timeline refers to the duration from the initial consultation to the patient being deemed fit for surgery. In addition, we also aimed to gather information on the differences in the timeline between the last pre-anesthesia consultation and the surgery, the frequency of referrals to other specialties, postoperative complications, the length of hospital stay (in days), and in-hospital mortality. Furthermore, we developed a preliminary hospital profile specifically designed for individuals undergoing surgery.

## Materials and methods

### Design and protocols applied to each group

#### Retrospective cross-sectional observational study

**Figure 1:**
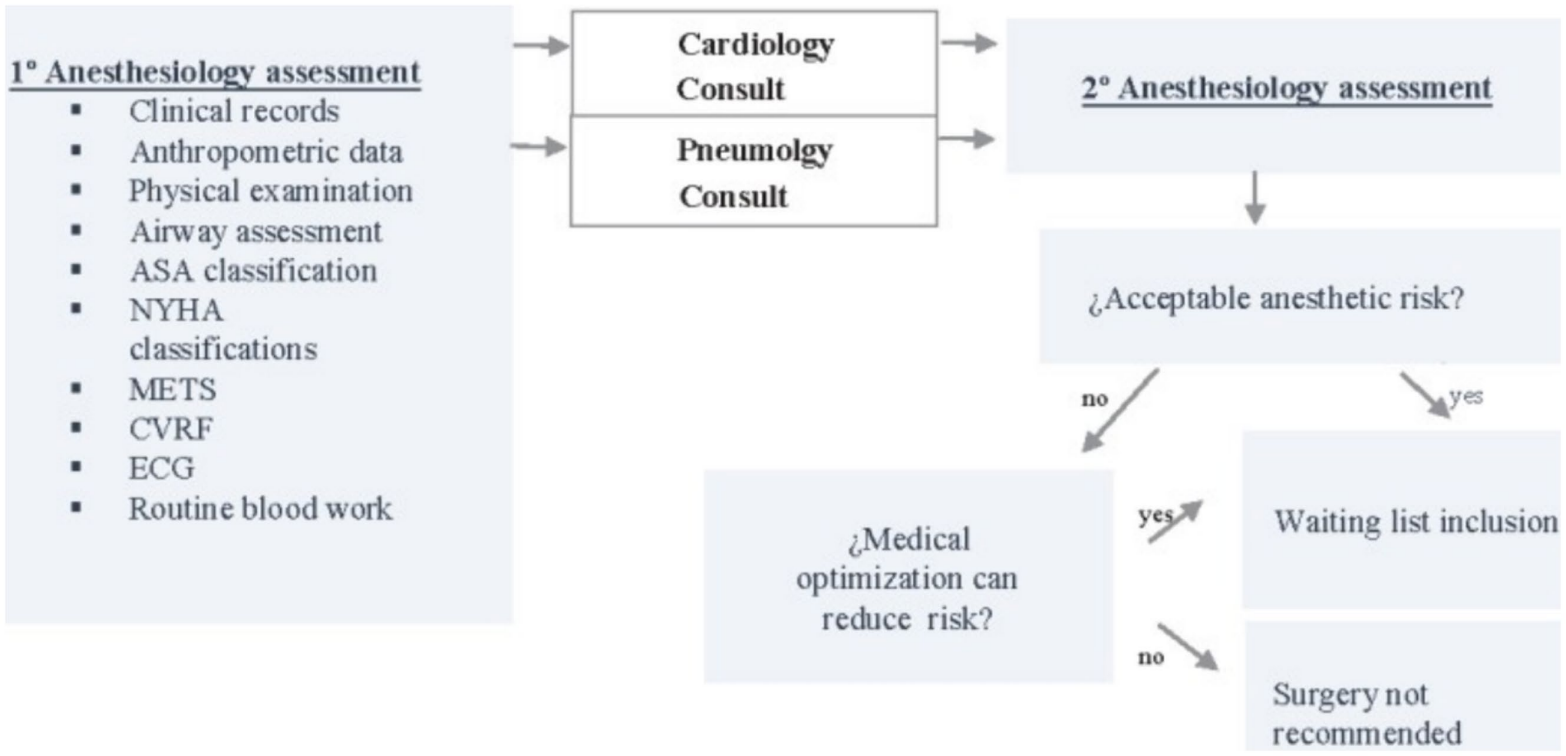
Group A algorithm. CV, Cardiovascular; CVRF, cardiovascular risk factors.

**Figure 2:**
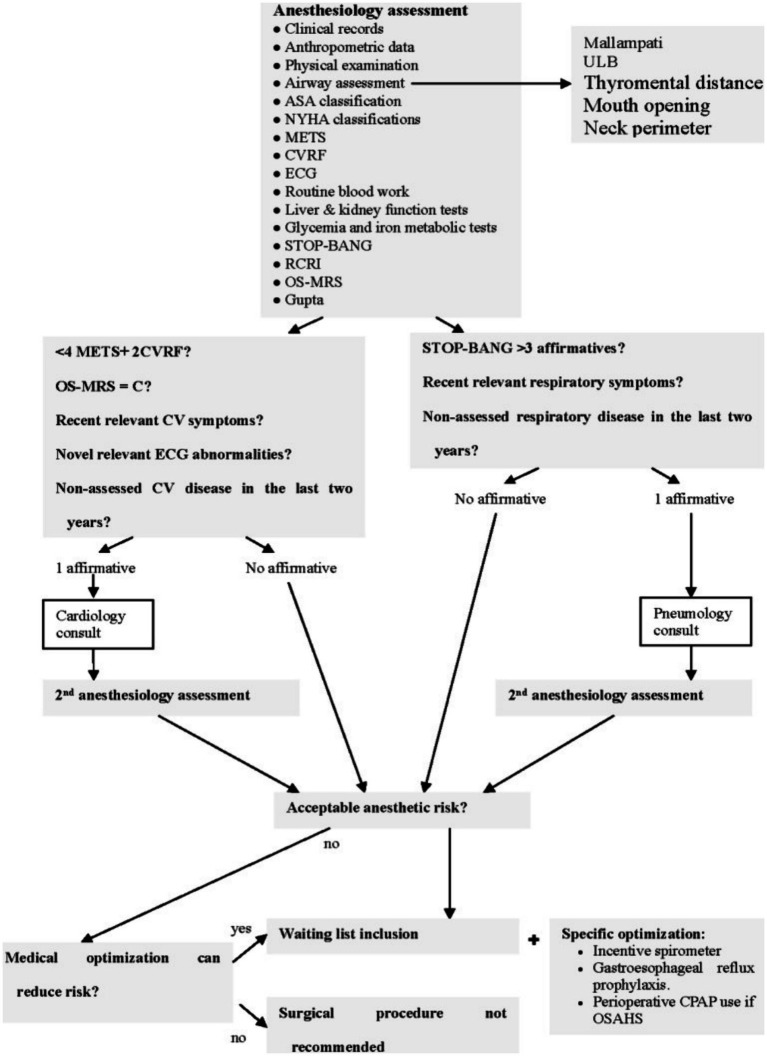
Group B algorithm.

The analysis was performed using the clinical records (CDs) of two different groups: Group A included 73 patients treated from January 2011 to December 2014, who underwent classical pre-anesthesia consultations (Figure 1), while group B included 133 patients treated between January 2015 and May 2022, who underwent the specific protocol proposed during pre-anesthesia consultations (Figure 2). A lack of relevant data caused the exclusion of two patients in group A and eight in group B.

### Population and sample

In this study, 206 adult patients (over 18 years of age) who underwent bariatric surgery in the period between 2011 and 2022 were included. All of them had consultations and surgeries performed at the University Hospital of Salamanca in the city of Salamanca, Castile and León, Spain.

Patients who required reoperation, who died while being on the waiting list, and who had incomplete surgery reports were excluded.

Given the evidence (21–25), which indicated a 28-day reduction in the intervention group (where the proposed protocol was utilized) compared to the non-standardized pre-anesthesia consultation group, we deemed that a sample size of 65 patients per group was necessary, assuming homoscedasticity and a 95% confidence interval. To account for an estimated patient loss of 7%, a total of 70 patients per group were suggested.

### Information collected

Information was gathered from each patient’s clinical history, including previous comorbidities such as obesity, arterial hypertension, and diabetes mellitus.

Information was also obtained about the main instruments for measuring patient severity, including New York Heart Association (NYHA), American Society of Anesthesiologists (ASA), and the Obesity Surgery Mortality Risk Score (OS-MRS).

The New York Heart Association (NYHA) Functional Classification is a functional scale of heart failure (HF), which provides a simple scoring system for the documentation of the severity of symptoms and can be used to assess responses to the treatment of heart failure (HF).

The ASA physical status classification system is used for assessing the physical status of patients before surgery. The current ASA classification is based on five groups:

- ASA I: A patient in normal health, healthy.
- ASA II: A patient with mild systemic disease and no functional limitation. Smokers, patients with controlled arterial hypertension (AHT), or patients with controlled diabetes mellitus (DM) may be in this ASA category.
- ASA III: A patient with severe systemic disease who has limited activity but no disability. This category may include patients with ischemic heart disease (angina or infarction) with exertional tolerance and chronic bronchitis with dyspnea on exertion.
- ASA IV: A patient with a disabling systemic disease that poses a continuing threat to life. ASA IV patients could include those with chronic bronchitis with dyspnea at rest and hemodialysis patients awaiting renal transplantation.
- ASA V: A moribund patient who does not expect survival beyond 24 h with or without surgery.

The OS-MRS is used to predict the risk of postoperative complications following bariatric surgery by stratifying patients into categories of (A) low risk, (B) intermediate risk, and (C) high risk, based on the presence of complications. To determine costs, we referred to Decree 25/2010 of 17 June, as published in the Official Gazette of Castilla y León (BOCYL), which outlines public prices for healthcare and services. These data allowed us to establish a relationship with each factor.

To assess delays in care, the primary variables considered included the duration, measured in days, from the initial consultation to when the patient became suitable for surgery. We also took into account the period, measured in days, from the last consultation to the surgery, and the number of referrals to specialist care. In addition, information about the length of hospital stay and in-hospital mortality was collected.

### Statistical analysis of the data

To describe the main variables, mean and standard deviation were used when the variable was numerical and frequency distribution was used when the variable was qualitative. To compare these variables between the two defined groups, the parametric Student’s *t*-test or Mann–Whitney U test was performed depending on whether the numerical variable had a normal distribution. The normality test used was the Shapiro–Wilks test. For the qualitative variables, the chi-squared test was performed.

The statistical analyses were performed using STATA/SE v16.0, and *p*-values below 0.05 were considered statistically significant.

## Results

The presented data revealed similarities in the demographics, anthropometrics, and clinical characteristics between the two groups, close to symmetric distribution (see Table 1). In terms of obesity-related health issues, group A had 39 (54.9%) patients with arterial hypertension, 26 (36.6%) with diabetes mellitus, and 37 (52.1%) with OSAHS, while group B had 68 (54.4%) patients with arterial hypertension, 56 (44.8%) with diabetes mellitus, and 75 (60%) with OSAHS. Furthermore, in group A, 53 (72.6%) patients had ASA III classification, 33 (46.5%) had an OS-MRS score of A, and 37 (52%) belonged to NYHA functional class 1; whereas in group B, there were 105 (78.9%) patients with ASA III classification and 51 (41.4%) with an OS-MRS score of A. Regarding the cost, the consultations for the patients in group A included 70 cardiology consultations, costing €33,880.78; 69 pulmonology consultations, costing €17,960.70; 71 initial anesthesia consultations, costing €16,180.19; and 48 follow-up anesthesia consultations, costing €6,623.52. In addition, there were 161.24 days in the critical care unit, costing €169,601.81, and 760 days of hospital stay, totaling €310,642.40. On the other hand, the patients in group B had 43 cardiology consultations, amounting to €20,812.43, and 66 pulmonology consultations, costing €17,179.80. Moreover, €28,030.47 was spent on the 123 initial anesthesia consultations, and €8,693.37 was spent on the follow-up anesthesia consultations. The resuscitation stay cost was €55,082.48 for 52.27 days and €447,408.32 for 1,168 days of hospital stay.

**Table 1:** Sample demographics.

Variables	Group A (*N* = 71)	Group B (*N* = 125)
Sex:		
Male	19 (28.8%)	42 (33.6%)
Female	52 (73.2%)	83 (66.4%)
Surgical history:		
No	29 (40.8)	35 (29.4%)
Abdominal surgery	7 (9.9%)	14 (11.8%)
Others	35 (49.3%)	70 (58.8%)
Prior cardiac surgery:		
No	63 (88.7%)	107 (85.6%)
Yes	8 (11.3%)	18 (14.4%)
Oncological history:		
No	69 (97.2%)	118 (94.4%)
Yes	2 (2.8%)	7 (5.6%)
Smoker:		
No	55 (91.6%)	87 (70.9%)
Yes	4 (6.7%)	30 (24.6%)
Former smoker	1 (1.7%)	5 (4.1%)
Snorter:	69 (100%)	119 (100%)
No	5 (7.2%)	16 (13.4%)
Yes	64 (92.8%)	103 (86.6%)
Size (cm)	162.92 ± 8.82	164.20 ± 10.36
Weight (Kg)	128.2 ± 22.32	127.81 ± 22.77
BMI (Kg/m)^2^	48.58 ± 7.13	47.23 ± 6.76
Age (Years)	46.30 ± 11.68	46.78 ± 10.32

A reduction in costs was observed in group B, both in the gross total and average per patient (Table 2). Comparing the groups A and B, the mortality rate decreased from 4.2 to 0.8%, and postoperative complications were 5.6% for group A and 2.4% for group B.

**Table 2:** Healthcare costs.

Variables	Group A (*N* = 71)	Group B (*N* = 125)	*p*-value
Gross cost	555.189.32€	577.204.87€	0.003
Mean per-patient cost	7819.56 ± 1.178.51€	4617.63€ ± 1.132.76€	0.000

Surgery time was longer (*p* < 0.001) in group A, with a mean duration of 266.18 min (+/− 108.27 SD), than in group B, with a mean duration of 167.39 min (+/− 93.44 SD). The frequency of referrals and the need and time for a follow-up anesthesia consultation showed improvements when the proposed algorithm was applied (Table 3).

**Table 3:** Observed perioperative changes.

Variables	Group A (*N* = 71)	Group B (*N* = 125)	*p*-value
Cardiology consults (referrals)	70 (98.6%)	43 (34.7%)	<0.001
Pneumology consults (referrals)	69 (97.2%)	66 (55.95%)	<0.001
Need for a second preanesthetic assessment	48 (71.6%)	63 (49.6%)	0.004
Time in PACU in min	3270.14 ± 9031.62	627.29 ± 644.73	0.016
Length of stay in days	10.86 ± 14.27	6.55 ± 37.11	0.575
Time on the waiting list in days	75.89 ± 74.99	81.97 ± 68.76	0.719
Time between the first and second preanesthetic assessment (days)	118.85 ± 158.45	26.95 ± 64.121	<0.001

## Discussion

The proposed protocol included a comprehensive airway examination, so data on thyromental distance, mouth opening, and the upper lip bite (ULB) test were not available for group A. The addition of neck circumference > 43 cm, ULB grade C, and Mallampati classification III or higher helped to more clearly define the predictors of difficult intubation (DI) in patients with obesity (51–53).

A reduction in the likelihood of encountering difficult intubation (DI) in pre-anesthesia was reported, without any increase in the actual DI. Although there is no gold standard for the assessment of the airway and neck circumference considered to be at risk of DI, the proposed algorithm appears to be effective in this regard (31, 51, 52, 54, 55). As for OSAHS, screening with the STOP-Bang questionnaire, diagnosis, and treatment with CPAP are recommended and adopted in this protocol (15, 56–58). Although they are time-consuming, it is worth implementing, considering the results obtained in this study. However, there is still debate regarding the appropriate cut-off point for an increased risk of postoperative complications.

The ASA (American Society of Anesthesiologists) and OS-MRS scores were similar in both groups, meaning a similar risk of postoperative complications, and within the incidence described in other studies (24, 59–63). Both groups had a mean BMI value of above 40 kg/m^2^, with group A at 48.58 kg/m^2^ and group B at 46.78 kg/m^2^, which fit the criteria for extreme obesity and bariatric surgery (64, 65). Cardiac disease was described in 11.3% of the patients in group A and 14.4% in group B, and the frequencies of arterial hypertension and OSAHS were concurrent with the findings of similar studies (66–68). Laparoscopic tubular gastrectomy was the most common technique, as is the trend observed in the literature (69, 70), and the reduction in the operative time may be attributed to the learning curve in the surgical team (71) as no statistical differences in complications were found. Postoperative nausea and vomiting were more frequent in group A, although still within the expected range; the paralytic ileus found may have been due to the use of opioids during and after the surgery (72, 73). The absence of expected respiratory complications in group B may be attributed to patient optimization according to the proposed protocol, which included CPAP and incentive spirometer, and its known benefits in this regard (15, 74). The difference in the mortality rate in favor of group B may be due to patient optimization and better performance of the surgical technique as the risks and preconditions of both groups were similar, as mentioned above.

The significant difference in the referrals to cardiology or pulmonology and the time to second anesthesia consultation, as well as the observed reduction in the length of stay in the resuscitation unit, are in favor of the application of the algorithm. The same is true for the fact that, although not statistically significant, the length of hospital stay was also shorter in group B, as its related costs affected the total cost of medical care. The cost per patient went from 7819.56€ (+/− 1178.51 SD) in group A to 4617.63€ (+/− 1132.76 SD) in group B (*p* < 0.005).

A more thorough review of the clinical records in group B, according to the protocol, and the other data losses in group A that were included for the assessment of patients in group B (e.g., the airway scores mentioned and history) may account for a larger number of records of smoking and previous surgeries. On the other hand, although the larger sample size in group B was mainly due to a longer recruitment period, the data showed congruence with the characteristics of group A.

## Conclusion and clinical implications

This study found a significant reduction in the time and costs of the pre-anesthetic assessment, as well as in unnecessary referrals to other specialties, the length of stay in the resuscitation unit, and the cost of care per patient. It was also able to demonstrate the benefits of a systematic approach to pre-anesthetic assessment in patients with obesity, as a thorough assessment of cardiovascular, respiratory, and airway conditions can lead to fewer complications, shorter hospital stays, reduced surgery time, and lower mortality rates. The proposed algorithm facilitates the optimization of patients so that they can undergo bariatric surgery in the best possible conditions.

### Limitations

Despite the differences in groups A and B in terms of size, we consider that the groups met the comparability criteria. The difference between the groups had minimal statistical impact.

## Data Availability

The raw data supporting the conclusions of this article will be made available by the authors, without undue reservation.
